# Characterization and Quantification of Heavy Metals in Oued Sebou Sediments

**DOI:** 10.1155/2019/7496576

**Published:** 2019-06-26

**Authors:** Abdelaiz Dra, Abdelali El Gaidoumi, Karim Tanji, Aziz Chaouni Benabdallah, Abdeslem Taleb, Abdelhak Kherbeche

**Affiliations:** ^1^Laboratory of Catalysis, Materials and Environment, Higher School of Technology, Sidi Mohamed Ben Abdellah University of Fez, 30000 Fez, Morocco; ^2^Laboratory, Engineering of Water and Environment, Department of Process Engineering and Environment, Faculty of Sciences and Technology of Mohammedia, Morocco

## Abstract

The discharge of large quantities of industrial and domestic effluents into the estuaries, with or without treatment, has led to an increase in the amount of micropollutants present in the sediments. In this study, we have assessed the quality of sediments of Sebou river studying the physicochemical parameters, percentage of organic matter, mineralogy, and trace levels of metal elements trapped in the sample sediments of Sebou river. The sediments samples were collected from the upstream of Fez river, confluence between the Fez river and the Sebou river, Ain Nokbi river, and edge of Sebou river, where wastewaters from the city of Fez are discharged. The sediments samples were characterized by scanning electron microscopy, X-ray diffraction, and Fourier Transform Infrared Spectroscopy, while trace levels of metallic elements, Calcium, Zinc, Copper, Cadmium, Iron, and Nickel, were determined by the ICP-AES analysis. The obtained results show that there is a significant change in the values of the studied metals which is probably due to industrial effluents. Indeed, the metal content in the sediments reaches particularly high values exceeding the limit recommended by WHO. These results suggested that the pollution by metallic industrial effluents discharged without treatments poses potential threat to the receiving rivers and may represent a danger for humans which are exposed to pollutants due to the numerous uses of such river waters.

## 1. Introduction

The metal elements traces (EMT) are most dreaded of the polluting substances owing to the fact that they are not biodegradable and the phenomenon of car purification proves generally to be unable to solve the problem [1]. And even if the sediments are quasi ultimate traps for the EMT, nevertheless this fixing is not final because of the change of the physicochemical conditions [2]. However, the majority of the legislations governing the acceptable levels of an element trace metal in a compartment of the environment (water, the biocoenosis, and the sediment or ground) refer to the total concentration rather than with the chemical shape of this element and the substrate in which it is [3]. This data does not provide any information about becoming element traces metal concerning its interaction in deposits, its biodisponibility, or its toxicity. The abundance of a metal element traces in a fraction of the sediment compared to another determines the impacts on a given medium [4].

Heavy metal pollution is an issue that concerns all communities affected by the preservation of water supplies at a certain level of quality. The examination of heavy metals in sediments contributes to the identification, understanding, and forecast of metal pollution. This contribution is even more important because this subset can, according to its nature and environmental conditions, act as a reservoir in the case of a massive input, which turns into a source of endogenous pollution if the discharge conditions are favorable for its use [5–9]. Given the magnitude of the latter, a series of investigations were carried out in aquatic ecosystem [5].  Table 1 summarizes some results of metal contamination of Moroccan and international rivers.

The pollution of Sebou river (“Oued Sebou”, Morocco) by heavy metals gradually accumulated in the sediments is the result of the metallic contamination of a stream of water [8, 10]. The Sebou river is the largest river in Morocco. In fact, the distance between its source in the Middle Atlas and the Atlantic is around 600 km. It plays a vital role in the provision of drinking water, irrigation, and industrial use throughout its watershed [11–13]. It can be divided into three distinct geomorphologic basins: the upper, middle, and lower Sebou. The sampling sites in this study are located in the middle of Sebou, where the river faces a very high pollution flow from the surface of the water and sediments; the city of Fez generates 40% of the total impact of water quality on the Sebou river; this city has 400 industrial units, employing more than 22,000 people and a wide variety of crafts. The objective of this work is to quantify and characterize the fraction of heavy metals accumulated in the sediments of Sebou river of Fez city [8].

## 2. Materials and Methods

### 2.1. Study Area

The studied area has a continental semiarid climate with cold winter and hot summer. The winter period, between October and April, is the rainy season, while the remaining months are mainly dry [10]. The base flow of the river is around 17 m^3^.

The geological characteristic of Sebou watershed includes clays and calcareous formations of the middle Atlas Mountains. Samples were selected along the Sebou river and its junction with Fez river, where the principal vector of pollution is noted [8]:

- Station N°1, “upstream of the Fez river”: located on the Fez river before its entry to the city, considered as a reference station.
- Station N°2, “Ain Nokbi”: located downstream of Fez city, representing the converging area of all the effluents from Fez city before joining the Sebou river.
- Station N°3, “confluence of Fez river with Sebou river”: the reference station which is not influenced by the pollutant emissions.
- Station N°4, “edge of Sebou river”: located on the Sebou river at approximately two kilometers downstream of the junction with Fez river.

### 2.2. Sampling and Conservation of Sediments

The sediments were collected by the manual coring method using a 1.5 m long and 15 cm diameter corer with a depth ranging from 5 cm to 15 cm. The samples were placed in decontaminated (24 h in HNO_3_) polyethylene containers and immediately conserved in a refrigerator (4°C).

### 2.3. Sediments Treatment

Total dissolution by acid attack consists of the most recommended treatment method for analyzing heavy metals [8]. 1.5 g of 80°C dried sample and 6 ml of hydrofluoric acid (HF 40%) were reflux heated at 100°C during 1 h in a 100 ml round-bottomed flask in order to dissolve silicate compounds [11]. Next, aqua regia (3 ml of nitric acid (HNO_3_ 65%) and 9 ml of hydrochloric acid (HCl 38%)) was added to this mixture with continuous reflux heating during 2 h [5]. The residue after the total attack was diluted in 100 ml of ultra pure water, filtered using a syringe equipped with a 0.45 filter *μ*m, and then analyzed by ICP-AES. The purpose of this analysis is measuring total concentration of trace elements considered as pollutants such as Ca, Zn, Cd, Fe, Cu, and Ni [11].

### 2.4. Methods of Analysis and Characterization

Measurements of the physicochemical parameters (temperature, pH, and electric conductivity (EC)) were performed in situ with a multiprobe (Probe Holder, Standard, portable hach) calibrated before each campaign [11].

The ICP-AES spectrometer used to measure the concentration of trace elements is Activa-M (Horiba Jobin Yvon, France) with argon plasma. The X-ray diffractometer used is X'Pert Pro Panalatycal equipped with an ultrafast X'Celerator scintillation detector with Cu K*α* radiation beam (*λ* = 0.154060 nm), operating at 40 kV voltage and 30 mA current with a copper target. Data in 2*θ* were collected between 10 and 80 degrees on powdered samples with a counting step of 0.02° and a counting time in 2 s steps. The Fourier Transformed Infrared Spectroscopy (FTIR) was used for the sediment samples for characterization in terms of functional groups. FTIR measurements were performed using a spectrometer (Vertex 70) and were carried out in transmittance mode, in the range of 400-4000 cm^−1^ with resolution of 4 cm^−1^ [12].

### 2.5. Determination of Organic Matter

The sediments were dried at 65°C in an oven for 24 h and calcined at 450°C for 1 h in a furnace. The loss on ignition corresponds to the mass difference between before and after calcination and represents the organic matter content [13].

## 3. Results and Discussions

### 3.1. Physicochemical Analyses of Sediment

#### 3.1.1. Measure of Temperature

Water temperature is an important factor in the aquatic environment because it regulates almost all physical, chemical, and biological reactions. In the study area, we noted that this temperature does not vary greatly from one station to another, Table 2, and remains close to the average annual temperature of the region, i.e., 21°C, with a minimum of 17.6°C and a maximum of 23.4°C [8].

#### 3.1.2. Measure of pH

The acidity decreases with high levels of organic matter and increases during low-water periods, when evaporation is high [8]. The pH values of the water in the alluvial groundwater of the Sebou (Table 2) do not show any significant variations, with a minimum of 7.73 (upstream of the Fez river) and a maximum of 8.9 (edge of Sebou) [14].

#### 3.1.3. Conductivity Measurement

Conductivity is one of the ways to validate physicochemical analyses of water. Indeed, contrasting measures on a medium make it possible to highlight the existence of pollution, mixing, or infiltration zones. This parameter also makes it possible to assess the quantity of salts dissolved in water. Sebou surface water is highly mineralized with conductivity values between 1014 *μ*s/cm and 1337 *μ*s/cm (Table 2). These values recorded in the rainfall return period could be attributed to precipitation that caused a dilution of water mainly due to subterranean flow water sources from the middle Atlas mountains [15–17].

#### 3.1.4. Organic Matter Determination

The calculation of the organic matter of the samples by calcination also shows the presence of high organic matter content in the different stations; the observed loss on ignition is attributed to the organic matter and therefore represents the mass percentage of organic matter [17]. The rate of organic matter varies from one station to another; it reaches a maximum rate of 10.36% upstream Fez river and a minimum rate of 5.81% on the edge of Sebou (Table 2). This variation is said to be related to the high pollutant load of the city of Fez and the development of phytoplankton in rivers [4].

### 3.2. Sediments Analysis by X-Ray Diffraction

**Figure 1:**
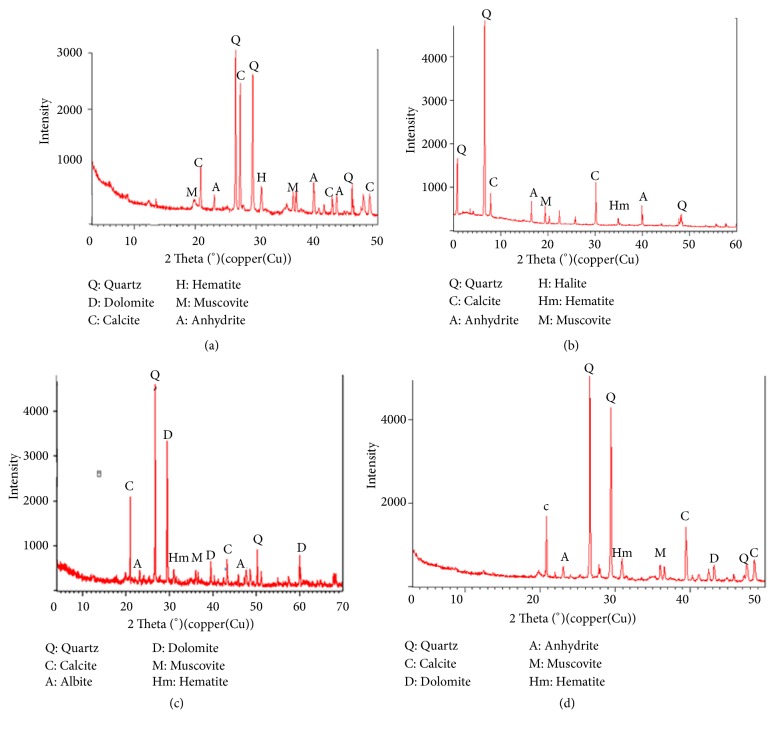
Mineralogical analysis by diffraction of x-rays of the sediments of various stations: (a) sample of the station N°1, (b) sample of the station N°2, (c) sample of the station N°3, and (d) sample of the station N°4.

**Figure 2:**
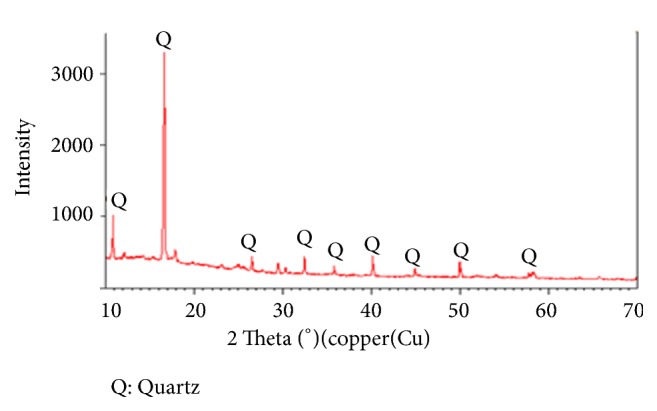
The diffraction spectrum of the attacked dry sediments from station N°1.

The mineralogical characterization of sediments is an important complement to physicochemical analyses. It makes it possible to identify different polluting ores or metals. The obtained information is based on the sediment X-ray diffractograms (Figure 1) which describes distinct crystalline phases: muscovite (KAl_3_Si_3_O_10_(OH)_2_), anhydrite (CaSO_4_), calcite (CaCO_3_), hematite (Fe_2_O_3_), halite (NaCl), quartz (SiO_2_), and dolomite CaMg (CO_3_)_2_ [18]. This mineralogy of sediment is a description of the mineralogical zone of the Sebou river. The diffractogram of the acid treated sediments of station N°1 (Figure 2) shows only the presence of quartz, indicating the attack of content phases [4, 17, 19].

### 3.3. Fourier Transform Infrared Spectroscopy

Analysis by IR spectroscopy makes it possible to identify absorption bands corresponding to the different vibrations of the characteristic bonds of the phases detected by XRD [18, 20].

**Figure 3:**
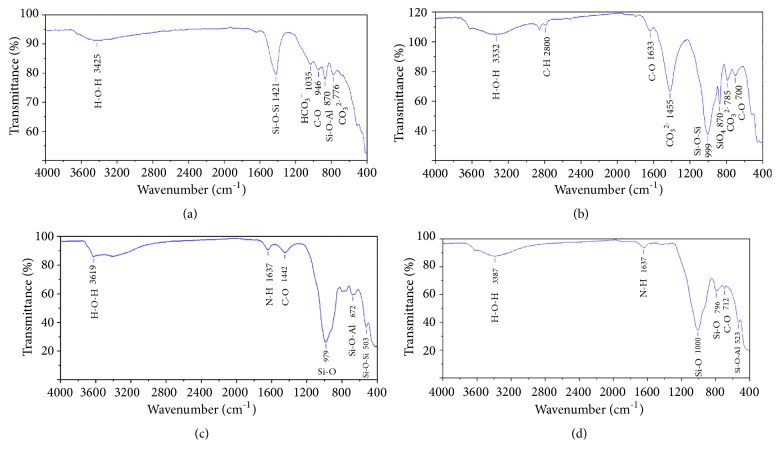
Sediments FTIR spectra of station N°1 (a); station N°2 (b); station N°3 (c); station N°4(d).

The IR spectrum of station N°1 sediments (Figure 3(a)) shows that bands centred at 776 and 870 cm^−1^ correspond to the vibration stretching of Si-O-Al and CO_3_^2−^, respectively, while the band at 946 cm^−1^ is attributed to the vibrations of the quartz Si-O-Si bonds. The H-O-H bonds are characterized by an intense band between 3425 cm^−1^ and centred around 1421 cm^−1^ corresponding to the valence vibrations of the Si-O-Si bond [2].

The IR spectrum of station N°2 sediments (Figure 3(b)) shows three characteristic bands of CO_3_^2−^: 1633 cm^−1^, 1455 cm^−l^, and 710 cm^−l^. The wide bands between 3332 cm^−1^ and 2800 cm^−l^ are assigned to the vibration of H-O-H and C-H cm^−1^. More intense band at 699 cm^−1^ is characteristics of silica. The bands at 3407 and 3619 cm^−1^ are attributed to the vibration of the O-H bond of hydroxyl groups [21]. The wide bands at 3332 and 1634 cm^−1^ are attributed to the axial and angular deformation of adsorbed water molecules [22].

Examination of the IR spectrum of sediments at station N°3 (Figure 3(c)) shows the following characteristic absorption bands: the 3619 cm-1 band measures the vibration of the O-H bond of the hydroxyl groups. The band at 979 cm -1 corresponds to the vibration of the Si-O bond. The 1442 cm-1 band is due to the vibrations of the C-O elongations. The vibration at 1637 cm-1 may be due to deformation of the N-H bond. The band 672 corresponds to the different modes of vibration of the Si-O-Al bond. The 503 cm-1 band is due to the vibration of the Si-O-Si bond.

The IR spectrum of station N°4 sediments (Figure 3(d)) reveals two bands of vibration at 796 cm^−1^ and 1000 cm^−1^ assigned to elongation of Si-O and Al-OH and bands at 523 cm^−1^ and 712 cm^−1^ characteristic of Si-O-Al and C-O. The spectrum represents also 3387 and 1637 cm^−1^ bands assigned to H-O-H and N-H [12, 18].

### 3.4. Sediment Analysis by Scanning Electron Microscopy

**Figure 4:**
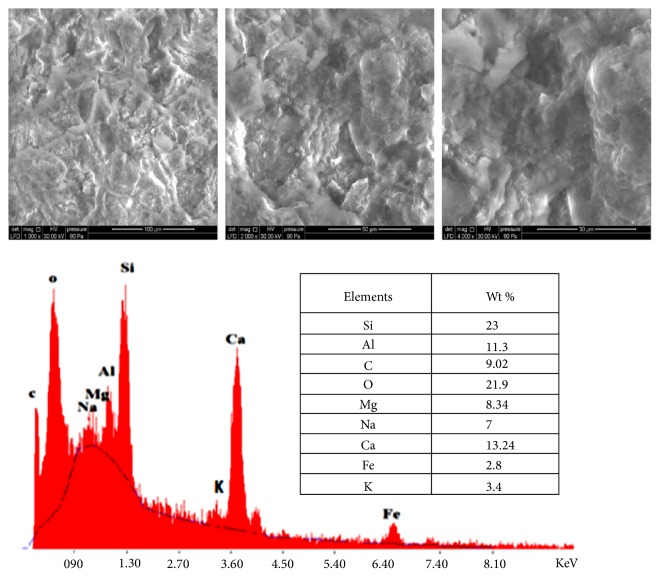
SEM images and EDX microanalysis spectrum of station N°1's sediments.

During the general observation of the sediments, it was found that there are grains of different morphologies after determining the chemical and mineralogical composition; we will try to assign the mineralogical constitution to each type of grain. The SEM analysis of sediments of studied stations showed similar results. Consequently, we chose the station N°1 as station model. SEM observation is coupled with quantitative chemical analysis by EDX, carried out on sediment grains (Figure 4). In accordance with the results obtained in DRX, the high proportion of iron, aluminium, calcium, and magnesium present in the sediments is related to the presence of either hematite hydroxide, calcium carbonate, dolomite CaMg(CO_3_)_2_, or muscovite KAl_3_Si_3_O_10_(OH) [6].

### 3.5. Chemical and Physicochemical Analyses of Industrial Effluents

#### 3.5.1. Analysis of Calcium

**Figure 5:**
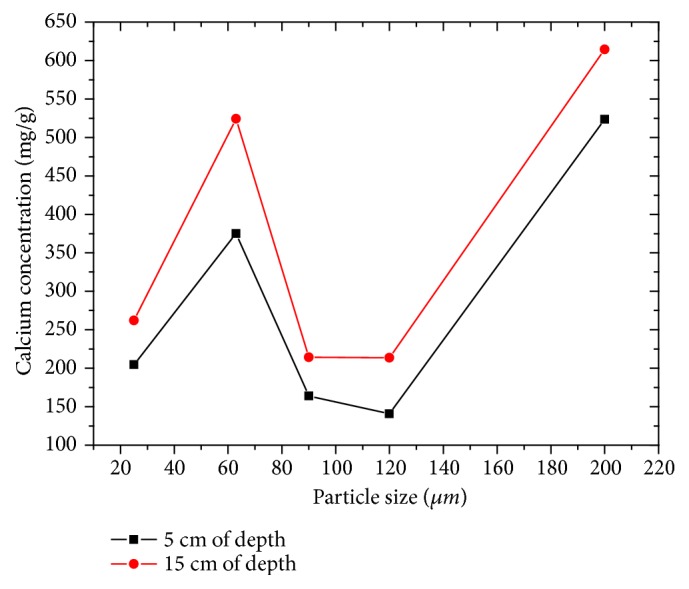
Evolution of calcium concentration as a function of particle size and depth.

The calcium ion concentration varies with particle depth as with particle size. The concentration increases with depth, so calcium is more concentrated at depth 15 cm than at depth 5 cm (Figure 5). The highest Ca content (614.6 mg/g) was recorded at the particle size of 200 *μ*m, while the lowest concentration was recorded at the particle size of 120 *μ*m (140.89 mg/g). The concentration of calcium ions is in direct relation to its geographical nature [8, 11].

#### 3.5.2. Analysis of Iron

**Figure 6:**
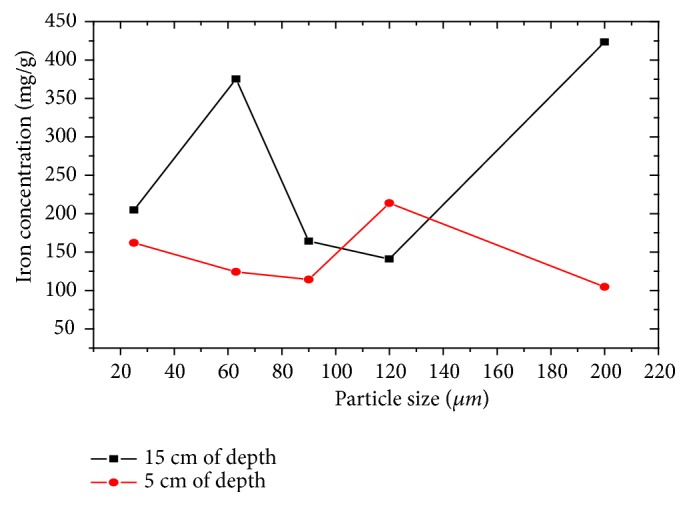
Evolution of iron concentration as a function of particle size and depth.

Figure 6 illustrates the evolution of iron concentration with granulometry (25-200 *μ*m) and depth (5 and 15 cm). The minimum level of iron concentration of 114.6 mg/g indicates that most of the inputs of this element probably come from the upstream part. The slight gradual enrichment towards the top of the river would be a sign of a recent influx of pollution. On the other hand, the depletion of iron levels towards the surface of the cores can be explained by the disappearance of natural or anthropogenic enrichment inputs. Overall, the iron contents recorded at the different levels of granulometry are very important [10, 23].

#### 3.5.3. Analysis of Copper

**Figure 7:**
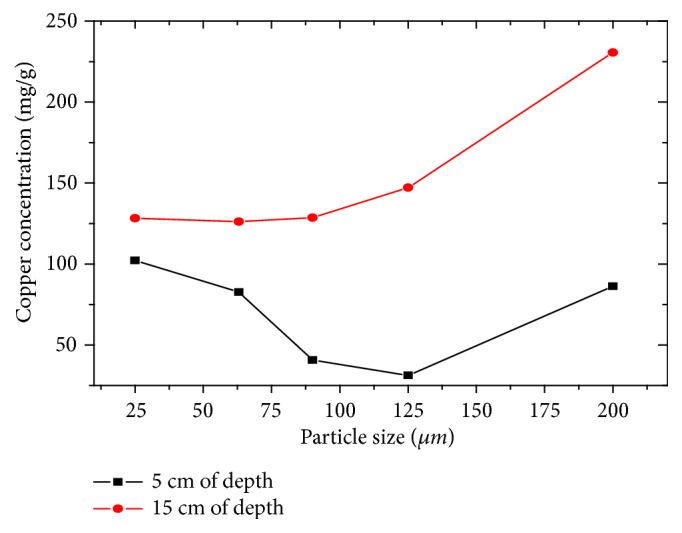
Evolution of copper concentration as a function of particle size and depth.

The average copper contents in the sediments of collected from top to bottom of the Sebou reservoir are 289 mg/g (depth of 15 cm and granulometry of 125 *μ*m), the upper value, and 27 mg/g (depth of 5 cm and granulometry of 200 *μ*m), the lower value (Figure 7). These values are very high comparing to the guide value of the copper contents of unpolluted sediments (< 25 *μ*g/g) [5], with the exception of the 5 cm depth with 125 *μ*m granulometry which has a relatively low content of 30.57 mg/g; this allows it to be classified as moderately contaminated sediments. The significant enrichment of the copper content at the depth of the 15 cm surface slice of the carrots on the deferential granulometries shows the existence of a recent contribution of the discharges which are strongly loaded with copper [4, 23, 24].

#### 3.5.4. Cadmium

**Figure 8:**
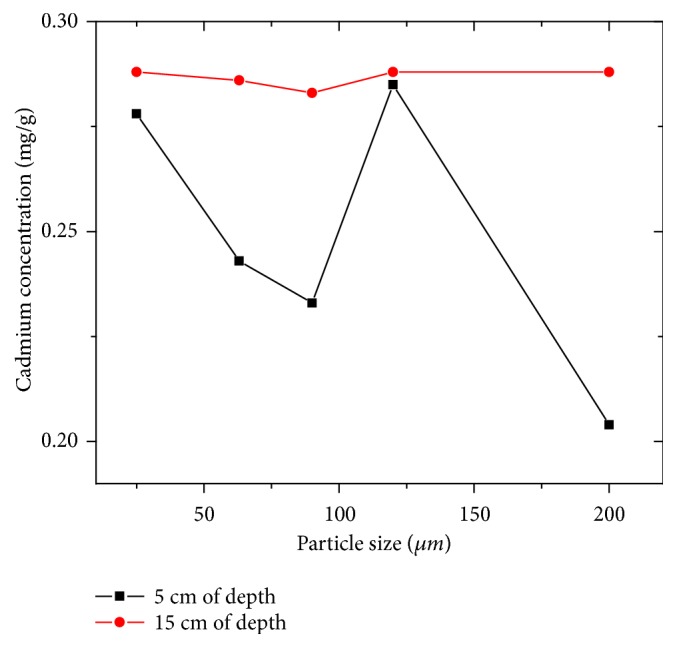
Evolution of cadmium concentration as a function of particle size and depth.

According to the obtained results (Figure 8), the main highest cadmium content was around 0.28 mg/g recorded at the granulometry of 125 *μ*m and depths of 5 cm and 15 cm, while the minimum content is about 0.204 mg/g at the granulometry of 200 *μ*m and depth of 5 cm. The average contents remain higher than the indicative value of 60 *μ*g/g (Table 3), which allows them to be classified as heavily polluted sediments; this result can be explained by the manufacture of Cd-Ni batteries, in the protection of steel against corrosion (cadmium plating), or as a stabilizer for plastics and pigments [4].

#### 3.5.5. Nickel

**Figure 9:**
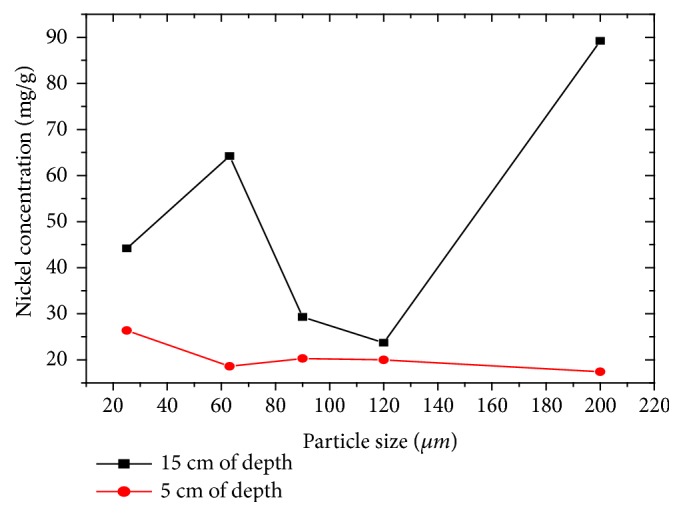
Evolution of nickel concentration as a function of particle size and depth.

Nickel concentration in the granulometries ranging from 25 to 200 *μ*m varies from 64.1 to 89.2 mg/g, respectively (Figure 9). These concentrations are greatly exceeding the guide value of unpolluted sediments (50 *μ*g/g) [5]. This content could be due to the following factors: the manufacture of steels and special alloys; surface coating by electrolysis; hydrogenation of oils and organic substances; the manufacture of paints [8, 12].

#### 3.5.6. Zinc

**Figure 10:**
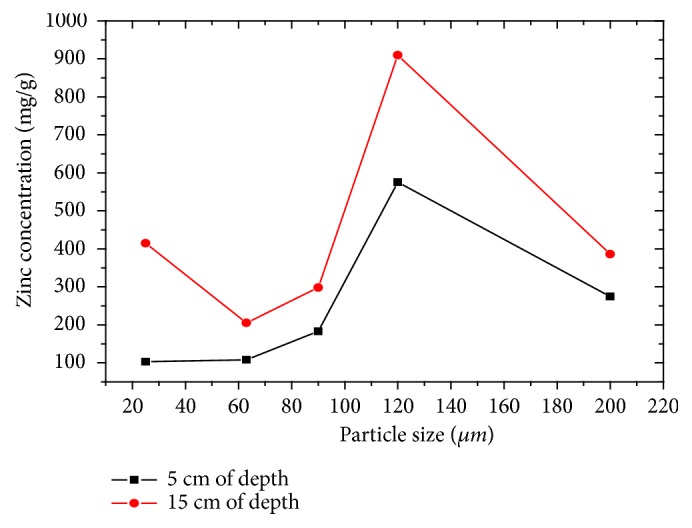
Evolution of zinc concentration as a function of particle size and depth.

Zinc contents are higher in the different granulometries for all studied depths (Figure 10), respectively, between 575.6 mg/g and 909.97 mg/g, except for the granulometries of 25 *μ*m and 63 *μ*m at depth of 5 cm, which is a low content, which allows it to be classified as heavily polluted sediments [25].

## 4. Conclusions

This work was interested in the study of the physicochemical characterization of sediments of Sebou river. The results showed that the levels of pollution by metals (Ca, Fe, Cu, Cd, Zn, and Ni) are high in all the studied stations (upstream of the Fez river, Ain Nokbi, confluence of Fez river with Sebou river, and edge of Sebou river) and exceed the recommended thresholds. This contamination highlights the negative impact of discharges from the city of Fez on the sediments and of course the water of Sebou river which is used in numerous utilizations. The protection of this water face to metal pollution is necessary and imperative so that it is still useful without risk of contamination.

## Figures and Tables

**Table 1:** Maximal metal trace contents in Moroccan and international rivers.

Authors	River, location	Layer	Pb	Zn	Cr	Ni	Cu
		(cm)	(*μ*g/g)	(*μ*g/g)	(*μ*g/g)	(*μ*g/g)	(*μ*g/g)
Singh et al. (2005) [9]	Gomti, India	Surface	40.3	41.7	8.15	15.7	5.0
Mohiuddin et al. (2010) [26]	Tsurumi, Japan	0-10	41	381	103	37	133
Suresh et al. (2011) [27]	Ponnaiyar, India	Surface	85.2	182.9	87.3	29.5	81.8
Lesven et al. (2010) [20]	Deule, French	0-6	2.490	5	-	28.333	179
Hassimi et al. [26]	Sebou, Morocco	0-10	431.2	157.7	1404	24.59	137.5
Hassimi et al. [26]	Fez city, Morocco	0-10	284.4	136.5	1376	120.25	81.9

**Table 2:** The physiochemical parameters and the percentage of organic matter of sediments.

Stations	Upstream of the Fez river	Ain Nokbi	Confluence of Fez and Sebou rivers	Edge of Sebou
Temperature [°C]	17.6	19	22	23.4
pH	7.73	8.27	8.19	8.9
Conductivity *μ*s /cm	1032	1014	1203	1337
Sediments Loss on ignition	5.81	6.30	6.26	10.36

**Table 3:** Guiding values for metal contents in sediments (*μ*g/g) proposed by USEPA [5].

Metal	Unpolluted material	Moderately polluted	Heavily polluted
Cd	< 0.1	0.1- 0,2	> 60
Cu	< 25	25−50	> 50
Pb	< 40	40−60	> 60
Zn	< 90	90−200	> 200

## Data Availability

No data were used to support this study.
